# Predictive modeling to evaluate long-term treatment effectiveness of darvadstrocel in patients with complex perianal fistulas in Crohn’s disease

**DOI:** 10.1186/s12876-024-03513-3

**Published:** 2024-12-30

**Authors:** Chitra Karki, Gary Hantsbarger, Erika Turkstra, Elisabetta Fenu, Ken Genenz, Inmaculada Gilaberte, Julián Panés

**Affiliations:** 1 Takeda Pharmaceuticals, Cambridge, MA USA; 2 Parexel International, Uxbridge, UK; 3 Takeda Pharmaceuticals, Zurich, Switzerland; 4 Takeda Pharmaceuticals, Madrid, Spain; 5 Formerly Gastroenterology Department, Institut d’Investigacions Biomèdiques August Pi i Sunyer (IDIBAPS), Centro de Investigación Biomédica en Red de Enfermedades Hepáticas y Digestivas, Hospital Clínic de Barcelona, Barcelona, Spain

**Keywords:** Crohn’s disease, Perianal fistula, Darvadstrocel, Stem cell therapy, Outcome measures, Real-world data, Pooled analysis, Clinical trial, Predictive modeling

## Abstract

**Background:**

Current therapies for complex Crohn’s perianal fistulas (CPF) have a limited ability to achieve long-term healing. Darvadstrocel (DVS) is an expanded allogeneic adipose-derived mesenchymal stem cell therapy that has demonstrated efficacy in treating complex CPF in clinical trials. There are, however, limited long-term comparative data with standard of care (SoC). The aim of this study was to combine clinical trial data and real-world evidence using statistical methodologies to predict long-term effectiveness of DVS versus SoC in patients with CPF.

**Methods:**

Data were pooled from a clinical trial (ADMIRE-CD) and two retrospective chart review studies (INSPECT and PREFACE). Predictive statistical models extrapolated clinical outcomes beyond observed follow-up using parametric curves, which were implemented into a semi-Markov model to obtain the number of patients in remission. The setting was multinational and multicenter. ADMIRE-CD was conducted in 49 hospitals in 7 European countries and Israel. INSPECT used data from the ADMIRE study. PREFACE involved patients from Belgium, France, Germany, Italy, and Spain. The participants were patients with complex CPF treated with DVS or SoC. Times to remission and relapse (clinical, and clinical plus patient-centric remission) were analyzed. Additionally, the proportion of patients in clinical and patient-centric remission was examined.

**Results:**

In total, 513 patients were included in the analysis (ADMIRE-CD [*N* = 200] and PREFACE [*N* = 313]). Patients in ADMIRE-CD and PREFACE were similar in age (median [interquartile range, IQR], 36 [20.0] versus 36 [22.0] years, respectively) and gender (males, 54% and 52%, respectively). The median (IQR) duration of Crohn’s disease was 9.4 [11.3] years for patients in ADMIRE-CD and 6.5 [12.9] years for patients in PREFACE. The estimated time to remission was shorter for patients treated with DVS versus SoC. The estimated time to relapse was longer for patients treated with DVS versus SoC. A higher estimated proportion of patients treated with DVS versus SoC had clinical and patient-centric remission at 24 months (48% and 35%, respectively) and 48 months (49% and 32%, respectively).

**Conclusion:**

This novel approach enabled pooled data from a clinical trial and real-world settings to predict long-term effectiveness of DVS versus SoC in patients with complex CPF.

**Supplementary Information:**

The online version contains supplementary material available at 10.1186/s12876-024-03513-3.

## Background

Perianal fistulas are a debilitating complication of Crohn’s disease (CD), which can be managed successfully in many cases [1, 2]. Complex Crohn’s perianal fistulas (CPF), however, are particularly challenging to treat and have a high relapse/recurrence rate [2, 3]. Current medical treatments for complex CPF include antibiotics, immunomodulators, and anti-tumor necrosis factor (anti-TNF) therapy (e.g. infliximab) [2, 4]. Infliximab is the only biologic treatment that has shown efficacy in a randomized controlled trial in patients with CPF [5–7]. The European Crohn's and Colitis Organisation and the American Gastroenterological Association guidelines support its use as a monotherapy, unlike antibiotics and immunomodulators, which are not recommended as monotherapies for the treatment of CPF [4, 7]. However, success rates with any of these therapies are limited [4, 8]. Studies are currently underway to evaluate the efficacy of guselkumab (phase 3) and ustekinumab (phase 4), versus placebo, in the treatment of CPF [9, 10].

Surgical interventions (e.g. seton placement, advancement flaps, and ligation of the intersphincteric tract) may be required in combination with pharmacological therapies to control disease or when patients are refractory to medical therapy [2, 11]. Combined therapy for CPF is associated with higher rates of healing than single therapy; however, complete healing, often defined as the absence of draining fistulas, is only achieved in up to 50% of patients [11–13]. Furthermore, the healing may only be short-term [8]. Both persistent CPF and surgical intervention may damage sphincters and cause fecal incontinence [14–16]. There is consequently an unmet need for interventions that provide long-term sustained remission in complex CPF and maintain patient quality of life.

Mesenchymal stem cells (MSC) are a novel treatment for complex CPF owing to their anti-inflammatory and immunomodulatory properties, and their efficacy and safety in patients with CPF have been demonstrated in several studies [2, 17–20]. Darvadstrocel (DVS, Cx601, Alofisel®) is a suspension of expanded allogeneic adipose-derived stem cells administered as a single dose; the efficacy and safety profile of which were demonstrated in the pivotal phase 3, randomized, double-blind, placebo-controlled 24- and 52-week study and 104-week follow-up, ADMIRE-CD (NCT01541579; initial registration 21/02/2012) [21–23]. The long-term effectiveness of DVS in patients treated in the ADMIRE-CD study has been assessed up to 156 weeks in a European chart review study, INSPECT [24].

Recently, a real-world evidence (RWE) study, PREFACE (a retrospective, non-interventional, chart review study), evaluated standard of care (SoC) treatment patterns in patients with CD with a new episode of complex CPF [25]. In this study, surgical drainage and seton placement were performed in a majority of patients and anti-TNF usage during CPF episodes was lower than expected.

Although DVS has been evaluated in ADMIRE-CD and INSPECT, there are limited long-term effectiveness data for DVS compared with SoC. In studies where long-term data are yet to be accrued, statistical modeling can be used to examine data for a given period of time and then extrapolate those data for an extended period of time. Statistical modeling can be used to predict long-term effectiveness of treatments by extrapolating data from clinical trials and RWE studies. We developed a statistical modeling approach synthesizing clinical trial data (ADMIRE-CD) and real-world data (INSPECT and PREFACE) to predict the long-term effectiveness of DVS versus SoC in patients with complex CPF (clinical trial number: not applicable).

## Methods

### Data sets

Time-to-event, anonymized patient data from three studies (ADMIRE-CD, INSPECT, and PREFACE) were used to create two data sets: 1) data from patients treated with DVS in ADMIRE-CD + INSPECT and 2) data from patients receiving SoC or placebo in ADMIRE-CD + INSPECT + PREFACE. SoC in PREFACE included seton placement, surgical drainage, anti-TNFs, antibiotics and immunosuppressants. Inclusion and exclusion criteria for ADMIRE-CD and INSPECT have been published previously [21, 22, 24]. Inclusion and exclusion criteria for PREFACE are described in Table S1. The results of the PREFACE study have not been published other than in abstract form; authors provided access to their data to support the current analysis. Data from ADMIRE-CD and INSPECT were combined because INSPECT contained patients who completed ≥ 52 weeks in the ADMIRE-CD trial. Identification numbers were used to link patient records between the two studies to prevent duplication. For patients with overlapping follow-up time, data from ADMIRE-CD were used as the primary source, with data from INSPECT being used after ADMIRE-CD follow-up had been completed. Patients participating in an interventional study were excluded from the PREFACE study.

The baseline values for ADMIRE-CD and PREFACE were assessed as part of a feasibility analysis to align variables and value formats (Tables S2 and S3). A full set of covariates was required for covariate-adjusted analyses, and missing values in the data sets were imputed using a multilevel Bayesian approach [26].

### Clinical outcome definitions

- Predictive parametric survival modeling was used to assess the following outcomes: time to clinical remission, time to clinical plus patient-centric (CPC) remission, time to clinical relapse from CPC remission, and time to CPC relapse from CPC remission (defined in Table 1).

**Table 1:** Clinical outcome definitions

Clinical outcome	Definition
Clinical remission	Clinically assessed closure of all EOs of the treated perianal fistulas that were draining at baseline despite gentle finger compression
CPC remission^a^	Clinically assessed closure of all EOs of the treated perianal fistulas that were draining at baseline despite gentle finger compression AND no occurrence of pain or discharge, measured by a score of 0 on the pain and discharge elements of the PDAI at the time of assessment (not baseline), as reported by patients
Clinical relapse from CPC remission	First loss of clinical remission status from first achievement of CPC remission
CPC relapse from CPC remission	First loss of CPC remission status from first achievement of CPC remission

*CPC* Clinical plus patient-centric, *DVS* Darvadstrocel, *EO* External opening, *PDAI* Perianal Disease Activity Index, *SoC* Standard of care

^a^CPC remission considers patient-assessed pain and discharge in addition to clinical remission and was used to calculate the estimated long-term effectiveness of DVS and SoC. CPC remission was suggested as a post-hoc analysis by clinical experts in countries where funding decisions for drugs are based on cost-effectiveness assessments such as the UK

- CPC remission considers patient-assessed pain and discharge in addition to clinical remission and was used to calculate the estimated long-term effectiveness of DVS and SoC. CPC remission was suggested as a post-hoc analysis by clinical experts in countries where funding decisions for drugs are based on cost-effectiveness assessments such as the UK. The number and proportion of patients in CPC remission at 24 and 48 months were determined by incorporating data from the predictive model into an extrapolation model to compare DVS with SoC for the treatment of complex CPF in CD (see ‘Analyses of the number of patients in remission’).

### Time-to-event analyses

- Remission and relapse outcomes were analyzed using a time-to-event longitudinal approach to incorporate and extrapolate the time-varying nature of the probability of achieving each clinical outcome. Within this framework, analyses focused specifically on achieving the first remission event since the start of ‘time at risk’. Once patients experienced an event (either a qualifying event or censoring event), they were removed from the analyses and did not provide additional time at risk in later analysis points. For time-to-remission analyses, the baseline visit was defined as the visit in which the treatment was initiated. A qualifying event was the first clinical or CPC remission event (Table 1). For time-to-relapse analyses, baseline time was defined as the first occurrence of clinical or CPC remission. A qualifying event was loss of clinical or CPC remission, defined as loss of clinical remission, or a score greater than 0 on the Perianal Disease Activity Index (PDAI) pain dimension, or a score greater than 0 on the PDAI discharge dimension. For all outcomes, a censoring event was a loss to follow-up, last-resort surgery (proctectomy), or death.

### Non-parametric survival (time-to-event) analyses

Non-parametric Kaplan–Meier (KM) analysis was performed for time-to-event outcomes. KM plots were generated for each endpoint and treatment arm. Differences between treatment arms were tested using the log-rank Mantel–Haenszel test [27]. Median event-free survival (i.e. the length of time during which the patient has no reoccurrence), mean restricted survival times, and respective 95% confidence intervals were estimated for each outcome by treatment group. Differences in survival times were tested using the log-rank (Mantel–Haenszel) and Wilcoxon (Gehan–Wilcoxon with Peto and Peto approximation) tests [28].

### Parametric survival (time-to-event) analyses

Predictive statistical models extrapolated clinical outcomes beyond the observed follow-up time using parametric curves fitted to the defined study populations for each treatment arm and outcome, as per the National Institute for Health and Care Excellence (NICE) Decision Support Unit Technical Support Document 14 [29]. Regression models were used to control for differences in baseline covariates, allowing exploration of the effect of patient demographic and disease characteristics on the outcomes and enabling prediction of covariate-adjusted outcomes to estimate population-adjusted treatment effectiveness. Before conducting covariate-adjusted analyses, comparisons between the ADMIRE-CD and PREFACE survival behaviors were assessed using KM analysis. All parametric models were fitted with a 4-week lead-in time to account for the absence of remission events recorded in ADMIRE-CD during this time.

The following parametric distributions were explored: Weibull, Gompertz, generalized gamma, log-logistic, and log-normal. Goodness of fit for all parametric models was evaluated by comparing the predicted curve to the KM curves and by comparing Akaike’s information criterion (AIC) and Bayesian information criterion (BIC) statistics. The best-fitting model for each outcome was selected by considering both clinical and face validity of the event generating mechanism and extrapolations, as well as statistical goodness of fit to the observed data.

The parametric survival models were assumed for the distribution of event times (T), by modeling the log-likelihood as:

$$\text{log-likelihood}\left(t, d, \alpha , \mu \right)=d\cdot \text{log}\left(h\left(t;\alpha ,\mu \right)\right)+\text{log}\left(S\left(t;\alpha ,\mu \right)\right)$$

in which *t* was the observed event time, *d* the censoring indicator (0 or 1), *μ* and *α* the location and ancillary distribution parameters, respectively, and *h(t)* and *S(t)* the distribution-specific hazard and survivor functions, modeled according to the probability distribution of survival times. The location parameter was assumed to be a linear function of the covariate effects, i.e. *μ* = *Xβ*. Covariates shared by ADMIRE-CD + INSPECT and PREFACE were included in the location and shape regression analysis. For the location parameter, all shared covariates were included (Table S4); however, for the shape parameter, some covariates were excluded owing to convergence issues or if they had a negligible effect on the shape parameter.

Study-specific effects (ADMIRE-CD + INSPECT vs PREFACE) were not included because it was assumed that long-term behavior, with everything else kept equal, would be the same for all studies. Including study-specific effects would significantly reduce the amount of information exchanged between the two data sets, therefore over-fitting each study shape.

### Analyses of the number of patients in remission

**Fig. 1:**
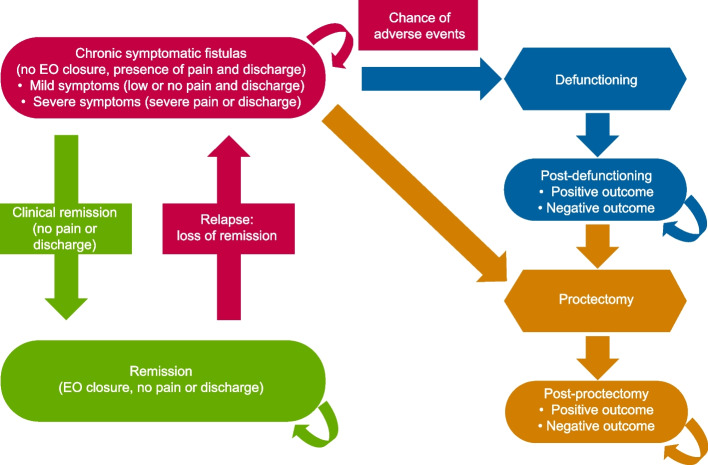
Semi-Markov model structure (extrapolation model) to conceptualize health states and probability of shift in health states in patients with CPF based on practice management. Because of the heterogeneity of the treatments received by patients, either as monotherapy or combinations, the interventions in the model were composed of treatment mixes, representing the average distribution of medical and surgical treatments received by a cohort of patients at any point in time. CPF, Crohn’s perianal fistulas; EO, external opening

The parametric survival models were incorporated into an extrapolation model to obtain the resultant number of patients in remission. The extrapolation model structure adopted was a semi-Markov model, which allowed an accurate description of the relapsing–remitting nature of the disease (Fig. 1), and was clinically validated by clinical experts to assess whether the outcomes reflected clinical practice. The extrapolation model included the following health states: remission health state, using the CPC definition of remission and chronic symptomatic fistulas (CSFs; initial health state), and including patients who did not achieve remission and/or are experiencing fistula-related symptoms of varying degrees. Patients were considered to enter the model in the CSF health state and receive initial treatment either with the current SoC or DVS in addition to SoC.

### Statistical models

A location- and shape-adjusted statistical model was chosen because it improved the fit and flexibility of the curves across clinical outcomes compared with the location-adjusted model. Covariate adjustments were integrated within the model, impacting the estimate of the location and/or shape parameters. Having the adjustment in both parameters provided higher flexibility for estimating survival curves over time.

### Clinical validation of the model

Five international clinical experts from England, France, the Netherlands, Scotland, and Sweden were interviewed to validate the structure, assumptions, and inputs of the semi-Markov model. Double-blind, 1:1 interviews were conducted via teleconference (experts were blind to the technology under assessment and companies involved, and investigators were blind to the names and institutions of clinicians). The interviews were blinded to avoid bias.

### Analysis software

Statistical analyses were performed in R [30]. The Bayesian models were fitted using *Stan* via the *RStan* R interface to the program [31, 32]. The Bayesian parametric model formulations were adapted based on the R package *survHE* [33].

## Results

### Patient disposition

In total, 513 patients were included in the analysis (Table 2).

**Table 2:** Patient disposition

Patients, n	ADMIRE-CD^a^	INSPECT^b^	PREFACE^c^	Pooled Analysis
DVS	101	43	0	101
Placebo/SoC	99	46	313	412
TOTAL	**200**	89	**313**	**513**^**d**^

The total number of patients in the ADMIRE-CD and PREFACE studies were 212 and 386, respectively. Patients were excluded from the analysis in this study if they did not have at least one completed clinical assessment after receiving DVS/placebo or after index date (*n* = 12 patients in ADMIRE-CD; *n* = 73 in PREFACE)

*CD* Crohn’s disease, *DVS* Darvadstrocel, *SoC* Standard of care, *TNF* Tumor necrosis factor

^a^Both groups received stable concomitant treatment (antibiotics, immunomodulators, anti-TNF, steroids) and the same surgical procedures of curettage of the fistula tract and closure of the internal opening

^b^Data from this study were combined with ADMIRE-CD data and not counted in the total pooled analysis patient population

^c^92% of all Crohn’s perianal fistulas in the full PREFACE population (386 patients) were treated with at least one surgical intervention; 87% were treated with at least one medical treatment

^d^Total pooled anonymized patient data

### Patient demographics and disease characteristics

Patients in the ADMIRE-CD and PREFACE studies were similar in age, gender, and CPF characteristics (number of internal openings [IOs] and external openings [EOs]). However, patients differed in characteristics such as median CD disease duration, prior biologic exposure, and moderate–severe luminal disease activity (Table 3). Compared with ADMIRE-CD, in PREFACE the median duration of CD was shorter and a lower proportion of patients had previously received antibiotics, biologics, and/or immunomodulators. Almost half (42%) of the patients in PREFACE had moderate or severe luminal disease, whereas all patients in ADMIRE-CD had mild/inactive disease. Patients in INSPECT were comparable to the overall ADMIRE-CD patient population for age, gender, weight, ethnicity, CD duration, and number of IOs and EOs.

**Table 3:** Baseline patient demographics and disease characteristics for patients enrolled in the ADMIRE-CD and PREFACE studies

	ADMIRE-CD *N* = 200	PREFACE *N* = 313	*p*-values
Age, years^a^			
Mean (SD)	38.2 (13.2)	37.9 (13.4)	0.804
Gender, *n* (%)			
Female	92 (46)	150 (48)	0.667
Male	108 (54)	163 (52)	
Ethnicity, *n* (%)			
Caucasian	184 (92)	307 (98)	0.001
Weight, kg^b^	*N* = 198	*N* = 291	
Mean (SD)	72.2 (14.8)	66.1 (14.3)	< 0.001
Duration of CD, years^c^	*N* = 199	*N* = 308	
Mean (SD)	11.4 (9.2)	9.4 (8.9)	0.015
Smoking status, *n* (%)	*N* = 39	*N* = 311	
Current smoker	17 (44)	106 (34)	
Former smoker	6 (15)	52 (17)	0.495
Never smoked	16 (41)	153 (49)	
Luminal disease, *n* (%)	*N* = 200	*N* = 293	
Mild/inactive^d^	200 (100)	169 (58)	
Moderate	0 (0)	106 (36)	< 0.001
Severe	0 (0)	18 (6)	
Number of IOs	*N* = 200	*N* = 294	
Mean (SD)	1.2^e^	1.3 (0.6)	Not calculable
Number of EOs	*N* = 197	*N* = 305	
Mean (SD)	1.5 (0.7)	1.5 (0.8)	1.000
Treatment, *n* (%)			
Previous antibiotic use	149 (74.5)	71 (23*)*	< 0.001
Previous biologic use	159 (79.5)	144 (46)	< 0.001
Previous immunomodulator use	155 (77.5)	213 (68)	0.023
CDAI	*N* = 198	*N* = 76	
Mean (SD)	90.7 (52.0)	172.9 (90.0)	< 0.001
CDAI ≤ 220	*N* = 200	*N* = 76	
n (%)	200 (100)	52 (68)	< 0.001
PDAI discharge score	*N* = 199	*N* = 85	
Mean (SD)	1.5 (0.9)	1.9 (1.0)	0.001
PDAI pain score	*N* = 199	*N* = 80	
Mean (SD)	1.2 (1.0)	1.8 (1.0)	< 0.001

*CD* Crohn’s disease, *CDAI* Crohn’s Disease Activity Index, *EO* External opening, *IO* Internal opening, *IQR* Interquartile range, *PDAI* Perianal disease activity index, *SD* Standard deviation

^a^Median (IQR): ADMIRE-CD, 36 (20.0); PREFACE, 36 (22.0)

^b^Median (IQR): ADMIRE-CD, 69.8 (16.2); PREFACE, 65 (20.0)

^c^Median (IQR): ADMIRE-CD, 9.4 (11.3); PREFACE, 6.5 (12.9)

^d^This category included patients in remission

^e^SD was not appropriate methodology here owing to the value of the IOs

### Unadjusted comparisons between ADMIRE-CD and PREFACE survival behaviors

**Fig. 2:**
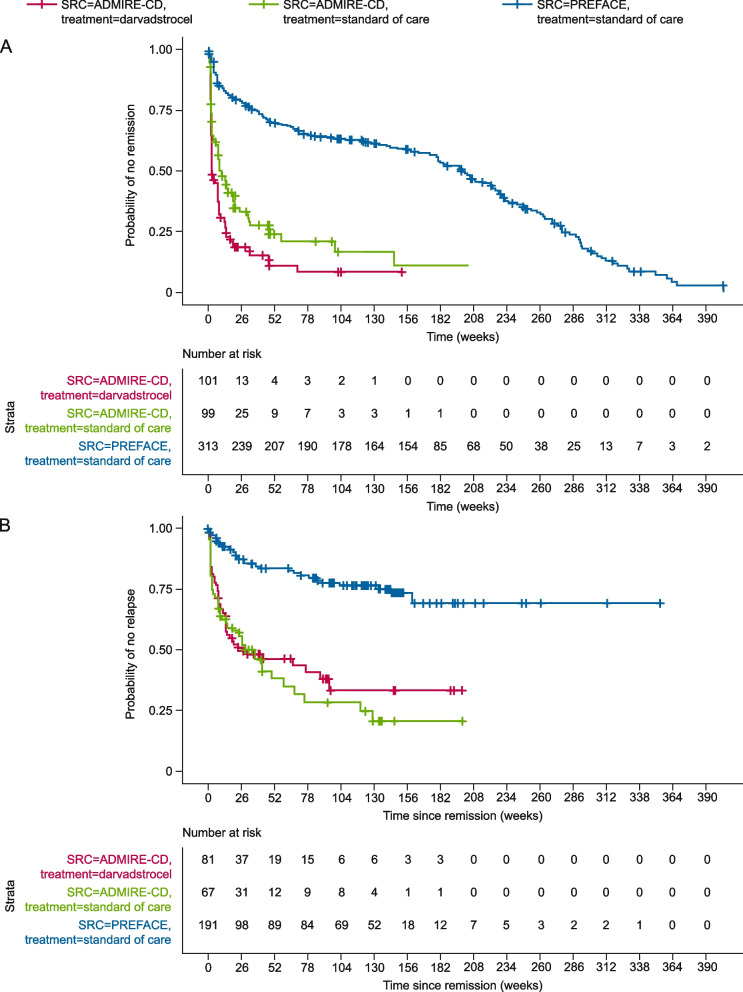
**A** Unadjusted comparison between data sets – time to CPC remission. **B** Unadjusted comparison between data sets – time to clinical relapse from CPC remission*.* The graphs depict ‘no response’ and ‘no remission’, therefore a curve with higher values indicates that the patient group has a lower chance of remission or relapse than the other groups and is therefore more stable. CD, Crohn’s disease; CPC, clinical plus and patient-centric; SRC, source

Despite a higher proportion of patients in PREFACE than ADMIRE-CD having moderate and severe luminal disease, PREFACE had substantially fewer remission and relapse events than ADMIRE-CD, indicating that its population comprised more stable patients (i.e. neither improving nor worsening) than ADMIRE-CD (Fig. 2A–B).

### Statistical models analysis and selection

For the statistical modeling, the parametric model that fitted the data best was chosen following discussions with external clinical experts. The model chosen differed between the outcomes. The best parametric models for time-to-remission and time-to-relapse outcomes were log-logistic and Weibull models, respectively. The Gompertz model was chosen as the parametric model that adjusted for location and shape because its estimates were considered appropriate and clinically plausible (i.e. estimated event rates falling within expected results based on the available body of evidence) by the panel of clinical experts. In addition, the Gompertz model predicted the 52-week probability of remission at visual assessment more accurately than the generalized gamma and log-normal models (data not shown).

For time to CPC remission, based on the AIC and BIC, the two best-fitting statistical models were the generalized gamma and log-normal models. For time to clinical relapse from CPC remission, all models resulted in similar fits; however, the long-term extrapolations differed between them. The Gompertz model indicated sustained remission for patients who had been in remission for about 4 years (data not shown). The AIC and BIC values for each goodness-of-fit parametric model for time to remission and time to clinical relapse from CPC remission are shown in Tables S5A and S5B, respectively.

### Clinical outcomes

#### Time to clinical remission and CPC remission

**Fig. 3:**
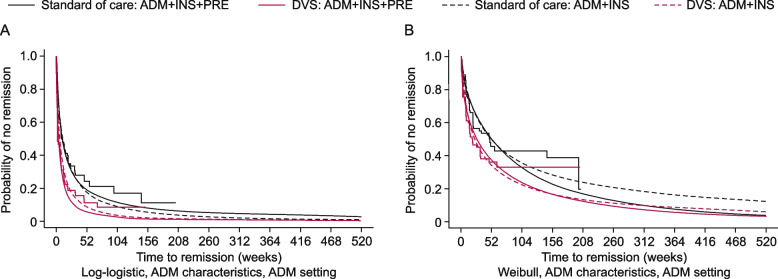
**A** Time to clinical remission – location and shape-adjusted model. **B** Time to CPC remission. ADM characteristics/settings means that the model uses the baseline characteristics/demographics of ADMIRE-CD not the PREFACE study. ADM + INS + PRE (solid lines) includes data from ADMIRE-CD, INSPECT, and PREFACE studies. ADM + INS (dashed lines) includes only data from ADMIRE-CD and INSPECT studies, using similar statistical methodology as the ADM + INS + PRE analysis. Red lines (solid or dashed) indicate results from patients treated with DVS. Black lines (solid or dashed) indicate results from patients treated with SoC. Smooth curves represent results of fitting parametric models. Stepped lines represent non-parametric Kaplan–Meier estimates. The best fit was used for each of the models; a different model, therefore, was used for graph A. ADM, ADMIRE-CD; CD, Crohn’s disease; CPC, clinical plus and patient-centric; DVS, darvadstrocel; INS, INSPECT; PRE, PREFACE; SoC, standard of care

The estimated time to clinical remission and CPC remission was shorter for patients receiving DVS than SoC when data were combined from all three studies. The inclusion of SoC data from PREFACE in the predictive models resulted in a shorter estimated time to clinical remission (Fig. 3A) and limited impact on time to CPC remission (Fig. 3B) for patients treated with DVS (solid red lines) when compared with the ADMIRE-CD + INSPECT data (dashed red lines). It also resulted in a longer estimated time to clinical remission (solid black line in Fig. 3A) and a shorter estimated time to CPC remission for patients treated with SoC (solid black line in Fig. 3B) when compared with the ADMIRE-CD + INSPECT data (dashed black lines).

#### Time to clinical relapse from CPC remission and CPC relapse from CPC remission

**Fig. 4:**
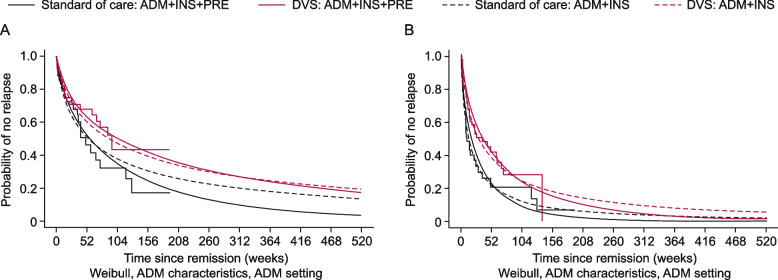
**A** Time to clinical relapse from CPC remission. **B** Time to CPC relapse from CPC remission. ADM characteristics/settings means that the model uses the baseline characteristics/demographics of ADMIRE-CD not the PREFACE study. ADM + INS + PRE (solid lines) includes data from ADMIRE-CD, INSPECT, and PREFACE studies. ADM + INS (dashed lines) includes only data from ADMIRE-CD and INSPECT studies, using similar statistical methodology as the ADM + INS + PRE analysis. Red lines (solid or dashed) indicate results from patients treated with DVS. Black lines (solid or dashed) indicate results from patients treated with SoC. Smooth curves represent results of fitting parametric models. Stepped lines represent non-parametric Kaplan–Meier estimates. ADM, ADMIRE-CD; CD, Crohn’s disease; CPC, clinical plus and patient-centric; DVS, darvadstrocel; INS, INSPECT; PRE, PREFACE; SoC, standard of care

The estimated time to clinical relapse from CPC remission and CPC relapse from CPC remission was longer for patients treated with DVS than for patients treated with SoC when using ADMIRE-CD + INSPECT data (dashed lines in Fig. 4). When additional SoC data from all patients in PREFACE were incorporated into the model (solid lines in Fig. 4), the estimated time to clinical relapse from CPC remission was unaffected in patients treated with DVS (red lines in Fig. 4A) and shorter in patients treated with SoC compared with ADMIRE-CD + INSPECT data (black lines in Fig. 4A). There was limited impact on the estimated time to CPC relapse from CPC remission (Fig. 4B), up to approximately weeks 52 and 104 for SoC and DVS, respectively, after which time to CPC relapse from CPC remission for both DVS and SoC decreased in the ADMIRE-CD + INSPECT + PREFACE data set compared with the ADMIRE-CD + INSPECT data set. In the ADMIRE-CD + INSPECT + PREFACE data set, the estimated time to clinical relapse from CPC remission was longer for patients treated with DVS than for patients treated with SoC (solid lines in Fig. 4A); patients receiving DVS had a delay in CPC relapse from CPC remission compared with those receiving SoC (solid lines in Fig. 4B).

#### Proportion of patients in CPC remission

**Fig. 5:**
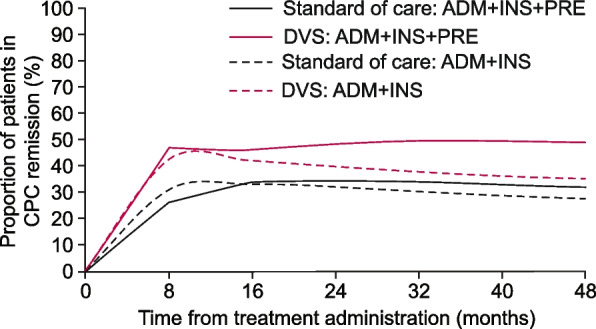
Differences in the percentage of patients in CPC remission treated with DVS and SoC 48 months after treatment. Estimated from semi-Markov models and adjusted for differences between data sets and bias from ADMIRE-CD and INSPECT. Bias adjustment was for the differences in patient inclusion in INSPECT compared with overall ADMIRE-CD patient population. These estimates are based on the Gompertz mode. ADM, ADMIRE-CD; CD, Crohn’s disease; CPC, clinical plus patient-centric; DVS, darvadstrocel; INS, INSPECT; PRE, PREFACE; SoC, standard of care

Using the extrapolation model and including data from ADMIRE-CD + INSPECT, a higher proportion of patients treated with DVS were estimated to have CPC remission at 24 months than patients who received SoC (39% and 32%, respectively) (Table 4 and Fig. 5). Including PREFACE data in the extrapolation model increased the predicted proportion of patients in CPC remission at 24 months in the DVS and SoC groups (48% and 35%, respectively) (Table 4 and Fig. 5). These estimated increases were maintained until 48 months (Fig. 5).

**Table 4:** Percentage of patients in CPC remission estimated from semi-Markov models, adjusted for the differences between data sets for ADMIRE-CD and INSPECT^a^

	ADMIRE-CD + INSPECT		ADMIRE-CD + INSPECT + PREFACE	
Month	DVS (%)	SoC (%)	DVS (%)	SoC (%)
0	0	0	0	0
8	43	31	47	26
24	39	32	48	35
48	35	27	49	32

*CD* Crohn’s disease, *CPC* Clinical plus patient-centric, *DVS* Darvadstrocel, *SoC* Standard of care

^a^Bias adjustment was for the differences in patient inclusion in INSPECT compared with overall ADMIRE-CD patient population. These estimates are based on the Gompertz model

## Discussion

In this study, predictive statistical models extrapolated clinical outcomes using combined data derived from clinical trials evaluating CPF treatment and a retrospective, non-interventional, chart review study to predict long-term effectiveness of DVS versus SoC in patients with complex CPF. This modeling approach has been used in other disease areas and enables us for the first time to predict long-term outcomes of DVS compared with SoC beyond the current durations seen in clinical trials and real-world studies [34, 35]. The parametric survival analyses that included real-world SoC data from the PREFACE study demonstrated that patients receiving DVS could potentially achieve earlier clinical and CPC remission and experience an increase in the time to clinical and CPC relapse from CPC remission than patients receiving SoC.

The Gompertz model was chosen as the base-case model because it was most similar to the disease trajectory observed after treatment in patients in a real-world setting. The predicted number of patients in CPC remission up to 3 years for SoC after incorporating PREFACE data was observed to reflect RWE, which provides confidence that the estimates are robust. Although the generalized gamma and log-normal models fitted the observed data reasonably well, they did not reflect real-world practice.

Semi-Markov modeling predicted that a higher proportion of patients treated with DVS would achieve CPC remission at 24 months than with SoC, and these differences were predicted to be maintained until 48 months after treatment, suggesting potential long-term maintenance of the disease-modifying effect of DVS.

Modeling predicted poorer long-term clinical outcomes for SoC than DVS in this study. Previous studies have reported a lack of long-term remission with other treatments or increased likelihood of relapse after cessation of therapy [36–39].

Single-dose DVS could promote sustained remission and may provide a treatment option for patients who are refractory to pharmacological treatments or in whom immunosuppression is contraindicated and could reduce the need for further surgical treatment. Additionally, DVS can be used in combination with standard-of-care pharmacotherapy in clinical practice. In ADMIRE-CD, patients in both the DVS and placebo groups received concomitant antibiotics, immunomodulators, anti-TNF, and steroids [21].

There was limited impact on time to CPC remission and time to clinical relapse from CPC remission in patients treated with DVS when comparing PREFACE data with ADMIRE + INSPECT data. The PREFACE study population had a shorter disease duration and lower prior exposure to biologics than the ADMIRE-CD + INSPECT population; both of these factors are associated with higher response rates to biologic drugs. This could account for the limited impact on time to CPC remission and clinical relapse from CPC remission.

Fewer remission and relapse events in PREFACE than in ADMIRE-CD suggested a more stable population compared with ADMIRE-CD. This may be due to ADMIRE-CD being a placebo-controlled trial, which typically include a more refractory patient population, or information bias from the PREFACE study, a real-world study in which patients were seen by clinicians during routine care or when they sought care (in clinical trials, the assessments are fixed). Inferences with respect to population stability need to be interpreted with caution. Strengths of this study include the pooling of clinical trial and RWE data, which allowed for greater statistical power and analysis of clinical outcomes in a rare disease for which real-world and long-term data are still accumulating. Another strength is the use of an extrapolation model validated by international clinical experts.

There are some limitations that need to be considered including the low number of events, particularly relapses; when compared with the number of parameters estimated, these led to dilution of information and a wide credibility interval. Predictions of clinical response outcomes at time points further from the point at which treatment was administered were particularly vulnerable to outlier effects owing to the small number of patients in the later phases of the trials. The simple functional form of the intertrial heterogeneity adjustment in the statistical models was relatively restrictive and implied that constant adjustment applied to all patients between trials. The proportion of patients with missing values in the SoC group (PREFACE) was greater than 30% for some variables. Therefore, the data should be interpreted with caution. A high proportion of missing PDAI data from the PREFACE study (73–74% versus 0.5% in ADMIRE-CD) could have introduced potential bias in the CPC remission/relapse analysis results. To mitigate this, a robust optimal imputation technique using a Bayesian approach was implemented to impute missing PDAI score categories by introducing dummy categories. No cross-study comparisons can be made with this study because it is the first of its type conducted in this patient population. According to a systematic review carried out by Fousekis et al. 2024*,* a limitation of DVS treatment may be its cost [40]. Evaluating cost-effectiveness was outside the scope of this study.

Data from this pooled analysis were collected prior to the completion of the global ADMIRE-CD II phase 3 study and so these data were not available for inclusion in the current analysis [41].

## Conclusion

DVS is the first allogeneic stem cell therapy indicated for the treatment of complex CPF. Our predictive modeling approach, using data derived from clinical trials and real-world settings, demonstrated that DVS was predicted to provide a shorter time to remission than SoC and a longer time to relapse than SoC, with a higher rate of CPC remission beyond 48 months than SoC. Further research is warranted to confirm the findings of this study.

## Supplementary Information

Supplementary Material 1: Table S1 Inclusion and exclusion criteria for PREFACE. Table S2 Imputation variables utilized in the covariate-adjusted models, categorical variables. Table S3 Imputation variables utilized in the covariate-adjusted models, numerical variables. Table S4 Covariates included in the location and shape regression analysis. Table S5A Goodness-of-fit measures, parametric models for time to CPC remission (AIC and BIC measure the goodness of fit of a model, penalized for the complexity of the model – lower numbers indicate better fit). Table S5B Goodness-of-fit measures, parametric models for time to clinical relapse from CPC remission (AIC and BIC measure the goodness of fit of a model, penalized for the complexity of the model – lower numbers indicate better fit).

## Data Availability

The data sets used and/or analyzed during the current study are available from the corresponding author on reasonable request.

## Abbreviations

ADM: ADMIRE-CD
AIC: Akaike’s information criterion
anti-TNF: anti-tumor necrosis factor
BIC: Bayesian information criterion
CD: Crohn’s disease
CPC: Clinical plus patient-centric
CPF: Crohn’s perianal fistulas
CSF: Chronic symptomatic fistula
DVS: Darvadstrocel
EO: External opening
INS: INSPECT
IO: Internal opening
IQR: Interquartile range
KM: Kaplan–Meier
MSC: Mesenchymal stem cells
NICE: National Institute for Health and Care Excellence
PDAI: Perianal Disease Activity Index
PRE: PREFACE
RWE: Real-world evidence
SD: Standard deviation
SoC: Standard of care
SRC: Source
TNF: Tumor necrosis factor

## References

[1] Schwartz DA, Ghazi LJ, Regueiro M. Guidelines for medical treatment of Crohn’s perianal fistulas: critical evaluation of therapeutic trials. Inflamm Bowel Dis. 2015;21(4):737–52. 25751068 10.1097/MIB.0000000000000377

[2] Aguilera-Castro L, Ferre-Aracil C, Garcia-Garcia-de-Paredes A, Rodriguez-de-Santiago E, Lopez-Sanroman A. Management of complex perianal Crohn’s disease. Ann Gastroenterol. 2017;30(1):33–44. 28042236 10.20524/aog.2016.0099 PMC5198245

[3] Panes J, Reinisch W, Rupniewska E, Khan S, Forns J, Khalid JM, et al. Burden and outcomes for complex perianal fistulas in Crohn’s disease: systematic review. World J Gastroenterol. 2018;24(42):4821–34. 30479468 10.3748/wjg.v24.i42.4821 PMC6235801

[4] Torres J, Bonovas S, Doherty G, Kucharzik T, Gisbert JP, Raine T, et al. ECCO guidelines on therapeutics in Crohn’s disease: medical treatment. J Crohns Colitis. 2020;14(1):4–22. 31711158 10.1093/ecco-jcc/jjz180

[5] Vasudevan A, Bruining DH, Loftus EV Jr, Faubion W, Ehman EC, Raffals L. Approach to medical therapy in perianal Crohn’s disease. World J Gastroenterol. 2021;27(25):3693–704. 34321838 10.3748/wjg.v27.i25.3693 PMC8291021

[6] Wetwittayakhlang P, Al Khoury A, Hahn GD, Lakatos PL. The optimal management of fistulizing Crohn's disease: evidence beyond randomized clinical trials. J Clin Med. 2022;11(11):3045. 10.3390/jcm11113045 PMC9181669 35683433

[7] Feuerstein JD, Ho EY, Shmidt E, Singh H, Falck-Ytter Y, Sultan S, et al. AGA Clinical Practice Guidelines on the Medical Management of Moderate to Severe Luminal and Perianal Fistulizing Crohn’s Disease. Gastroenterology. 2021;160(7):2496–508. 34051983 10.1053/j.gastro.2021.04.022 PMC8988893

[8] Rayen J, Currie T, Gearry RB, Frizelle F, Eglinton T. The long-term outcome of anti-TNF alpha therapy in perianal Crohn’s disease. Tech Coloproctol. 2017;21(2):119–24. 28066859 10.1007/s10151-016-1578-4

[9] Clinicaltrials.gov. A study of guselkumab in participants with fistulizing, perianal Crohn's disease (FUZION CD). https://clinicaltrials.gov/study/NCT05347095. Accessed on 24 Sept 2024.

[10] Clincaltrials.gov. USTekinumab in fistulising perianal Crohn's disease (USTAP). https://clinicaltrials.gov/study/NCT04496063. Accessed on 24 Sept 2024. vail.

[11] Lightner AL, Ashburn JH, Brar MS, Carvello M, Chandrasinghe P, van Overstraeten AB, et al. Fistulizing Crohn’s disease. Curr Probl Surg. 2020;57(11): 100808. 33187597 10.1016/j.cpsurg.2020.100808

[12] El-Gazzaz G, Hull T, Church JM. Biological immunomodulators improve the healing rate in surgically treated perianal Crohn’s fistulas. Colorectal Dis. 2012;14(10):1217–23. 22251452 10.1111/j.1463-1318.2012.02944.x

[13] Yassin NA, Askari A, Warusavitarne J, Faiz OD, Athanasiou T, Phillips RK, et al. Systematic review: the combined surgical and medical treatment of fistulising perianal Crohn’s disease. Aliment Pharmacol Ther. 2014;40(7):741–9. 25115149 10.1111/apt.12906

[14] van Praag EM, Stellingwerf ME, van der Bilt JDW, Bemelman WA, Gecse KB, Buskens CJ. Ligation of the intersphincteric fistula tract and endorectal advancement flap for high perianal fistulas in Crohn’s disease: a retrospective cohort study. J Crohns Colitis. 2020;14(6):757–63. 31696918 10.1093/ecco-jcc/jjz181 PMC7346888

[15] Singh B, Mc CMNJ, Jewell DP, George B. Perianal Crohn’s disease. Br J Surg. 2004;91(7):801–14. 15227686 10.1002/bjs.4613

[16] Alvarez JA, Bermejo F, Algaba A, Hernandez MP, Grau M. Surgical repair and biological therapy for fecal incontinence in Crohn’s disease involving both sphincter defects and complex fistulas. J Crohns Colitis. 2011;5(6):598–607. 22115381 10.1016/j.crohns.2011.06.004

[17] Kotze PG, Shen B, Lightner A, Yamamoto T, Spinelli A, Ghosh S, et al. Modern management of perianal fistulas in Crohn’s disease: future directions. Gut. 2018;67(6):1181–94. 29331943 10.1136/gutjnl-2017-314918

[18] de la Portilla F, Alba F, Garcia-Olmo D, Herrerias JM, Gonzalez FX, Galindo A. Expanded allogeneic adipose-derived stem cells (eASCs) for the treatment of complex perianal fistula in Crohn’s disease: results from a multicenter phase I/IIa clinical trial. Int J Colorectal Dis. 2013;28(3):313–23. 23053677 10.1007/s00384-012-1581-9

[19] Lee WY, Park KJ, Cho YB, Yoon SN, Song KH, Kim DS, et al. Autologous adipose tissue-derived stem cells treatment demonstrated favorable and sustainable therapeutic effect for Crohn’s fistula. Stem Cells. 2013;31(11):2575–81. 23404825 10.1002/stem.1357

[20] Lightner AL, Wang Z, Zubair AC, Dozois EJ. A systematic review and meta-analysis of mesenchymal stem cell injections for the treatment of perianal Crohn’s disease: progress made and future directions. Dis Colon Rectum. 2018;61(5):629–40. 29578916 10.1097/DCR.0000000000001093

[21] Panes J, Garcia-Olmo D, Van Assche G, Colombel JF, Reinisch W, Baumgart DC, et al. Expanded allogeneic adipose-derived mesenchymal stem cells (Cx601) for complex perianal fistulas in Crohn’s disease: a phase 3 randomised, double-blind controlled trial. Lancet. 2016;388:1281–90. 27477896 10.1016/S0140-6736(16)31203-X

[22] Panes J, Garcia-Olmo D, Van Assche G, Colombel JF, Reinisch W, Baumgart DC, et al. Long-term efficacy and safety of stem cell therapy (Cx601) for complex perianal fistulas in patients with Crohn’s disease. Gastroenterology. 2018;154(5):1334–42. 29277560 10.1053/j.gastro.2017.12.020

[23] Garcia-Olmo D, Gilaberte I, Binek M, Ajl DH, Lindner D, Selvaggi F, et al. Follow-up study to evaluate the long-term safety and efficacy of darvadstrocel (mesenchymal stem cell treatment) in patients with perianal fistulizing Crohn’s disease: ADMIRE-CD phase 3 randomized controlled trial. Dis Colon Rectum. 2022;65(5):713–20. 34890373 10.1097/DCR.0000000000002325 PMC8985696

[24] Panés J, Bouma G, Ferrante M, Kucharzik T, Nachury M, de de la Portilla Juan F, et al. INSPECT: A retrospective study to evaluate long-term effectiveness and safety of darvadstrocel in patients with perianal fistulizing Crohn’s disease treated in the ADMIRE-CD trial. Inflamm Bowel Dis. 2022;28(11):1737–45. 35099555 10.1093/ibd/izab361 PMC9629463

[25] Ferrante M, Siproudhis L, Poggioli G, Reinshagen M, Milicevic S, Roset M, et al. P801 Treatment patterns of complex perianal fistula in Crohn’s disease in five European countries: the PREFACE study, a retrospective chart review. J Crohns Colitis. 2020;14(Supplement_1):S628.

[26] Gelman A, Carlin JB, Stern HS, Dunson DB, Vehtari A, Rubin DB. Bayesian data analysis. 3rd ed. New York: CRC Press; 2013.

[27] Harrington DP, Fleming TR. A class of rank test procedures for censored survival data. Biometrika. 1982;69(3):553–66.

[28] Collett D. Modelling survival data in medical research. 3rd ed. New York: CRC Press; 2014.

[29] Latimer N. NICE DSU. Technical Support Document 14: survival analysis for economic evaluations alongside clinical trials - extrapolation with patient-level data, 2013. [cited 2022 July 05]. Available from: https://www.sheffield.ac.uk/nice-dsu/tsds/survival-analysis. 27905716

[30] R Core Team. R Foundation for Statistical Computing: Vienna, Austria. R: a language and environment for statistical computing, 2016. [cited 2022 July 07]. Available from: https://www.R-project.org/.

[31] Carpenter B, Gelman A, Hoffman MD, Lee D, Goodrich B, Betancourt M, et al. Stan: a probabilistic programming language. J Stat Soft. 2017;76(1):1–32. 10.18637/jss.v076.i01 PMC9788645 36568334

[32] Stan Development Team. RStan: the R interface to Stan, 2019. [cited 2022 July 13]. Available from: https://cran.r-project.org/web/packages/rstan/vignettes/rstan.html.

[33] Baio G. survHE: survival analysis for health economic evaluation and cost-effectiveness modelling. J Stat Soft. 2020;95(14):1–47.

[34] Antimicrobial Resistance Collaborators. Global burden of bacterial antimicrobial resistance in 2019: a systematic analysis. Lancet. 2022;399(10325):629–55. 35065702 10.1016/S0140-6736(21)02724-0 PMC8841637

[35] Marks C, Abramovitz D, Donnelly CA, Carrasco-Escobar G, Carrasco-Hernández R, Ciccarone D, et al. Identifying counties at risk of high overdose mortality burden during the emerging fentanyl epidemic in the USA: a predictive statistical modelling study. Lancet Public Health. 2021;6(10):e720–8. 34118194 10.1016/S2468-2667(21)00080-3 PMC8565591

[36] Tozer P, Ng SC, Siddiqui MR, Plamondon S, Burling D, Gupta A, et al. Long-term MRI-guided combined anti-TNF-alpha and thiopurine therapy for Crohn’s perianal fistulas. Inflamm Bowel Dis. 2012;18(10):1825–34. 22223472 10.1002/ibd.21940

[37] Sands BE, Anderson FH, Bernstein CN, Chey WY, Feagan BG, Fedorak RN, et al. Infliximab maintenance therapy for fistulizing Crohn’s disease. N Engl J Med. 2004;350(9):876–85. 14985485 10.1056/NEJMoa030815

[38] Domenech E, Hinojosa J, Nos P, Garcia-Planella E, Cabre E, Bernal I, et al. Clinical evolution of luminal and perianal Crohn’s disease after inducing remission with infliximab: how long should patients be treated? Aliment Pharmacol Ther. 2005;22(11–12):1107–13. 16305724 10.1111/j.1365-2036.2005.02670.x

[39] Molendijk I, Nuij VJ, van der Meulen-de Jong AE, van der Woude CJ. Disappointing durable remission rates in complex Crohn’s disease fistula. Inflamm Bowel Dis. 2014;20(11):2022–8. 25159455 10.1097/MIB.0000000000000148

[40] Fousekis FS, Mpakogiannis K, Lianos GD, Koukoudis A, Christodoulou DK, Papaconstantinou I, et al. Effectiveness and safety of darvadstrocel in patients with complex perianal fistulizing Crohn’s disease: a systematic review. Ann Gastroenterol. 2024;37(1):46–53. 38223244 10.20524/aog.2023.0850 PMC10785025

[41] Serclova Z, Garcia-Olmo D, Chen ST, Wexner S, Panés J, Wu C, et al. OP18 Efficacy and safety of darvadstrocel treatment in patients with complex perianal fistulas and Crohn’s Disease: results from the global ADMIRE-CD II phase 3 study. J Crohn’s and Colitis. 2024;18(Supplement_1):i34–5.

