# Supplementary material for: Predictive modeling to evaluate long-term treatment effectiveness of darvadstrocel in patients with complex perianal fistulas in Crohn’s disease
Source: BMC Gastroenterol. 2024 Dec 30;24:479. doi: 10.1186/s12876-024-03513-3 (PMC11684150; doi:10.1186/s12876-024-03513-3)
Supplement: Supplementary file 1 — Supplementary Material 1: Table S1 Inclusion and exclusion criteria for PREFACE. Table S2 Imputation variables utilized in the covariate-adjusted models, categorical variables. Table S3 Imputation variables utilized in the covariate-adjusted models, numerical variables. Table S4 Covariates included in the location and shape regression analysis. Table S5A Goodness-of-fit measures, parametric models for time to CPC remission (AIC and BIC measure the goodness of fit of a model, penalized for the complexity of the model – lower numbers indicate better fit). Table S5B Goodness-of-fit measures, parametric models for time to clinical relapse from CPC remission (AIC and BIC measure the goodness of fit of a model, penalized for the complexity of the model – lower numbers indicate better fit). [file 12876_2024_3513_MOESM1_ESM.docx]

**Supporting Information**

## Table S1 Inclusion and exclusion criteria for PREFACE

| Inclusion criteria | Exclusion criteria |
| --- | --- |
| - ≥18 years old at the time of treatment initiation - Documented diagnosis of complex CPF (as per the American Gastroenterological Association definition) - Treatment initiation for a new episode of complex CPF between September 2011 and September 2014 in Belgium, France, Germany, Italy, and Spain - Treatment initiation was defined as either first treatment after diagnosis or after a period of remission of at least 6 months - Data were collected for a minimum of 3 years after index date (defined as the date of initiation of treatment for a new episode of complex CPF during the eligibility period) or death - Treated mainly for perianal disease and not luminal disease according to physician’s criteria | - Participation in an interventional clinical trial during the follow-up period after initiation of treatment for complex CPF - Indeterminate or unspecified type of inflammatory bowel disease - Diagnosis of ulcerative colitis |

*CPF* Crohn’s perianal fistulas

## Table S2 Imputation variables utilized in the covariate-adjusted models: categorical variables

| Variable | Statistics/values ADMIRE-CD | Frequencies (% of valid) | |
| --- | --- | --- | --- |
|  |  | **ADMIRE-CD** | **PREFACE** |
| Smoking status | Current smoker  Former smoker  Never smoked | 108 (54.0)  32 (16.0)  60 (30.0) | 107 (34.2)  53 (16.9)  153 (48.9) |
| Vaginal fistula | 1. No | 200 (100) | 298 (95.2) |
|  | 2. Yes | 0 (0.0) | 15 (4.8) |
| Luminal disease status | Mild  Moderate  Severe | 200 (100)  0 (0.0)  0 (0.0) | 181 (57.8)  114 (36.4)  18 (5.8) |
| Gender | 1. Female | 92 (46.0) | 150 (47.9) |
|  | 2. Male | 108 (54.0) | 163 (52.1) |
| CDAI ≤220 | 1. No | 0 (0.0) | 76 (24.3) |
|  | 2. Yes | 200 (100) | 237 (75.7) |
| Previous antibiotic | 1. No | 51 (25.5) | 242 (77.3) |
|  | 2. Yes | 149 (74.5) | 71 (22.7) |
| Previous biologic | 1. No | 41 (20.5) | 169 (54.0) |
|  | 2. Yes | 159 (79.5) | 144 (46.0) |
| Previous immunomodulator | 1. No | 45 (22.5) | 100 (31.9) |
|  | 2. Yes | 155 (77.5) | 213 (68.0) |
| INSPIRE site^a^ | 1. No | 0 (0.0) | 93 (29.7) |
|  | 2. Yes | 200 (100) | 220 (70.3) |

Missing values in the data sets were imputed using a multilevel Bayesian approach. Dummy values were generated for categorical variables. Baseline characteristics were log-transformed if positive and scaled to have unit variance. A multivariate normal distribution was assumed between all baseline characteristics, with data set-specific means and common variance. All variables were included in the model. The model was run for 2000 iterations (1000 discarded for warm-up) for two chains. The data sets were unscaled, and the categorical variables reclassified based on the category with the greater average ranking per missing observation.

^a^The INSPIRE registry is a European observational, post-approval study designed to evaluate the real-world effectiveness and safety of DVS in patients with complex CPF for up to 36 months after DVS administration. Patients were enrolled in different sites.

*CD* Crohn’s disease, *CDAI* Crohn’s disease activity index, *CPF* Crohn’s perianal fistulas, *DVS* Darvadstrocel

## Table S3 Imputation variables utilized in the covariate-adjusted models: numerical variables

| Variable | ADMIRE-CD | PREFACE |
| --- | --- | --- |
| Weight, mean (SD) | 72.1 (14.8) | 65.8 (14.3) |
| Age, mean (SD) | 38.2 (13.2) | 37.9 (13.4) |
| Number of EOs, mean (SD) | 1.5 (0.7) | 3 (1.6) |
| Number of IOs, mean (SD) | 1.2^a^ | 1.2 (0.6) |
| Discharge PDAI score, mean (SD) | 1.5 (0.9) | 1.6 (1.6) |
| Pain PDAI score, mean (SD) | 1.2 (1.0) | 1.8 (1.7) |
| CD duration, years, mean (SD) | 11.5 (9.3) | 9.3 (8.8) |
| Hematocrit, mean (SD) | 8.4 (0.9) | 39 (7.2) |
| CDAI, mean (SD) | 90.4 (51.9) | 161.3 (106.3) |

^a^SD was not appropriate methodology here owing to the value of the IOs.

*CD* Crohn’s disease, *CDAI* Crohn’s disease activity index, *EO* External opening, *IO* Internal opening, *PDAI* Perianal Disease Activity Index, *SD* Standard deviation

## Table S4 Covariates included in the location and shape regression analysis

| Regression on location  parameter covariates | Regression on shape  parameter covariates |
| --- | --- |
| - Treatment (SoC vs DVS) - Weight - Age - Number of EOs - Number of IOs - Smoking status (current vs former vs never smoker) - PDAI discharge score (0–4) - PDAI pain score (0–4) - Fistula type (non-vaginal vs vaginal) - Luminal disease (mild vs moderate vs severe) - CD duration (years) - Hematocrit levels - Gender (female vs male) - CDAI score - CDAI score ≤220 (no vs yes) - Previous antibiotic (no vs yes) - Previous anti-TNF (no vs yes) - Previous immunomodulator (no vs yes) - INSPIRE^a^ site (no vs yes) - Study (ADMIRE-CD+INSPECT vs PREFACE) | - Treatment (SoC vs DVS) - Number of EOs - Number of IOs - Luminal disease (mild vs moderate vs severe) - CD duration (years) - Previous anti-TNF (no vs yes) - Previous immunomodulator (no vs yes) - INSPIRE^a^ site (no vs yes) |

^a^The INSPIRE registry is a European observational, post-approval study designed to evaluate the real-world effectiveness and safety of DVS in patients with complex CPF for up to 36 months after DVS administration. Patients were enrolled in different sites.

*CD* Crohn’s disease, *CDAI* Crohn’s disease activity index, *CPF* Crohn’s perianal fistulas, *DVS* Darvadstrocel, *EO* External opening, *IO* Internal opening, *PDAI* Perianal Disease Activity Index, *SoC* Standard of care, *TNF* Tumor necrosis factor

## Table S5A Goodness-of-fit measures: parametric models for time to CPC remission (AIC and BIC measure the goodness of fit of a model, penalized for the complexity of the model – lower numbers indicate better fit)

| Parametric model | AIC | BIC |
| --- | --- | --- |
| Generalized gamma | 1050.099 | 1063.600 |
| Log-normal | 1076.002 | 1086.128 |
| Gompertz | 1079.894 | 1090.020 |
| Log-logistic | 1085.236 | 1095.361 |
| Weibull | 1100.479 | 1110.605 |
| Exponential | 1148.842 | 1155.592 |

*AIC* Akaike’s information criterion, *BIC* Bayesian information criterion, *CPC* Clinical and patient-centric

## Table S5B Goodness-of-fit measures: parametric models for time to clinical relapse from CPC remission (AIC and BIC measure the goodness of fit of a model, penalized for the complexity of the model – lower numbers indicate better fit)

| Parametric model | AIC | BIC |
| --- | --- | --- |
| Log-normal | 490.1425 | 497.8974 |
| Weibull | 490.7000 | 498.4549 |
| Gamma | 491.0490 | 498.8039 |
| Log-logistic | 491.3465 | 499.1014 |
| Generalized gamma | 492.0107 | 502.3506 |
| Gompertz | 492.7804 | 500.5353 |
| Exponential | 496.0099 | 501.1798 |

*AIC* Akaike’s information criterion, *BIC* Bayesian information criterion, *CPC* Clinical and patient-centric
